# Seroprevalence and risk factors for *Toxoplasma gondii* infection in wild, domestic and companion animals in urban informal settlements from Salvador, Brazil

**DOI:** 10.1371/journal.pntd.0013303

**Published:** 2025-12-16

**Authors:** Leonela Bazan, Hernán Darío Argibay, Waléria Borges-Silva, Luís Fernando Pita Gondim, Thaís Auxiliadora dos Santos Mattos, Juliete Oliveira Santana, Eduardo Mendes da Silva, Michael Begon, Hussein Khalil, Federico Costa, Ianei de Oliveira Carneiro

**Affiliations:** 1 Institute of Biology, Federal University of Bahia, Salvador, Bahia, Brazil; 2 Institute of Collective Health, Federal University of Bahia, Salvador, Bahia, Brazil; 3 School of Veterinary Medicine and Animal Science, Federal University of Bahia, Salvador, Bahia, Brazil; 4 Gonçalo Moniz Institute, Oswaldo Cruz Foundation, Ministry of Health, Salvador, Bahia, Brazil; 5 Department of Evolution, Ecology and Behavior, University of Liverpool, Liverpool, United Kingdom; 6 Department of Wildlife, Fish, and Environmental Studies, Swedish University of Agricultural Sciences, Umeå, Sweden; 7 Yale School of Public Health, New Haven, Connecticut, United States of America; Independent Researcher, UNITED KINGDOM OF GREAT BRITAIN AND NORTHERN IRELAND

## Abstract

*Toxoplasma gondii* is a globally neglected zoonotic parasite, particularly prevalent in socioeconomically vulnerable areas. Various animal species serve as reservoirs for *T. gondii* across different regions, including domestic cats, livestock, and a variety of wild and synanthropic animals. In urban areas, especially informal settlements, the close coexistence of humans, domestic animals, and wildlife may influence local transmission dynamics. This study evaluated the seroprevalence and associated risk factors for *T. gondii* infection in domestic and synanthropic animals from two low-income neighborhoods in Salvador, Brazil. A cross-sectional study was conducted in the neighborhoods of Marechal Rondon and Pau da Lima from October 2021 to February 2023. Blood samples were collected from domestic animals (288 dogs, 112 cats, 27 chickens, and six horses) and synanthropic species (54 brown rats and 75 big-eared opossums). Serological tests were performed using an indirect immunofluorescence antibody test. Questionnaires were used to collect environmental, demographic, and socioeconomic data from households where sampling took place. Generalized linear mixed models were applied to identify predictors of exposure. Seroprevalence was highest in chickens (66.7%), followed by dogs (37.2%), rats (24.1%), cats (22.3%), opossums (20%), and horses (16.7%). No significant factors were found to be associated with *T. gondii* seroprevalence in chickens, horses, rats, or opossums. Nevertheless, in dogs and cats, homemade diets increased the odds of infection by nearly six times compared to commercial feeding. Dogs from Pau da Lima were twice as likely to be infected as those from Marechal Rondon. These findings underscore the importance of promoting safe pet management, improving sanitation, and monitoring sentinel species to mitigate zoonotic risks in urban informal settlements.

## Author summary

*Toxoplasma gondii* is a parasite that infects humans and animals worldwide, significantly impacting impoverished areas. In this study, we investigated the prevalence and factors associated with *T. gondii* infection in domestic and urban wildlife from two low-income neighborhoods in Salvador (BA, Brazil). We collected blood samples from dogs, cats, chickens, horses, rats, and opossums to test for antibodies against the parasite and analyzed environmental and lifestyle factors that might influence infection risk. Chickens showed the highest infection rates (67%), followed by dogs (37%), rats (24%), cats (22%), opossums (20%), and horses (17%). We found that homemade diets significantly increased the likelihood of infection in dogs and cats compared to commercial pet food.

Additionally, dogs from one neighborhood were twice as likely to be infected as those from the other, likely due to environmental conditions. These findings highlight how diet, environment, and urban living conditions affect the spread of *T. gondii*. By improving sanitation, promoting responsible pet care, and monitoring animals that share human environments, we can reduce the risk of this parasite in vulnerable communities.

## Introduction

*Toxoplasma gondii* (Nicolle and Manceaux, 1908) is an apicomplexan coccidian responsible for toxoplasmosis, a neglected zoonotic parasitic disease associated with poor living conditions [1,2]. Approximately 30% of the global human population shows evidence of exposure to *T. gondii* [3]; however, seroprevalence varies significantly worldwide [4]. In highly endemic regions, such as Brazil, it can reach nearly 90% in specific demographic groups [5]. *T. gondii* definitive hosts are members of the felid family, while all birds and mammals, including domestic and wild animals, serve as intermediate hosts [3,6]. *T. gondii* undergoes sexual reproduction in the intestines of felids, with oocysts excreted in the faeces [7]. Under suitable humidity and temperature, these oocysts become infective within 2–3 days and can persist in the environment, contaminating soil, water, and vegetation [8]. Oocysts are highly resistant and, under suitable humidity and temperature conditions, can remain infective in soil for up to 18 months [9] and in water for 18–54 months under experimental conditions [8,10]. A single felid can excrete millions of oocysts, leading to widespread contamination of soil, water, vegetables, gardens, and recreational areas [11]. *T. gondii* transmission occurs mainly through ingesting contaminated food or water and congenital transmission from mother to fetus during pregnancy [12].

Among the various zoonotic pathogens circulating in urban environments, *T. gondii* stands out for its complex life cycle, wide host range, and environmental persistence, which together facilitate its transmission across species and habitats. In informal urban settlements, where humans and animals coexist in close proximity and sanitation conditions are often precarious, the risk of environmental contamination with *T. gondii* is particularly high [2]. These characteristics make *T. gondii* an especially relevant sentinel pathogen for understanding the ecological interfaces between humans, domestic animals, and urban wildlife. Urban animals such as pets, poultry, rats, and opossums can serve as intermediate hosts for *T. gondii*, where the parasite multiplies asexually, forming tissue cysts in organs such as muscles and the brain. These intermediate hosts play a significant epidemiological role in human, animal, and environmental health. As they share the same environment and familiar sources of infection as humans, these animals can act as effective sentinels for monitoring *T. gondii* exposure [13–16].

As definitive hosts, domestic cats are the primary source of oocyst shedding in urban ecosystems [17]. Cats are mostly infected during the first months of life, with prevalence increasing in those with street access or those fed homemade or raw meat [18]. Other urban mammals and birds can also act as intermediate hosts and can become infected [12] Due to their coprophagic habits, tendency to roll in cat feces, and close contact with their owners, pet dogs can indicate human contamination risk [19,20]. Chickens are particularly effective indicators of oocyst contamination in soil, as they forage constantly and remain in close contact with the ground [21]. Synanthropic rodents indicate environmental contamination by *T. gondii* oocysts and are a primary source of infection for definitive hosts, playing a significant role in ecological dissemination [22,23].

Given the technical challenges in directly quantifying oocysts in the environment, assessing the serological status of *T. gondii* in free-ranging urban animals is a valuable proxy for evaluating environmental contamination and associated epidemiological risks to human populations [16,24,25]. Using urban animals as sentinels to monitor the spread of zoonotic pathogens is essential, as health risks are interconnected across species, and the emergence and persistence of these diseases are driven by complex, multidisciplinary factors [15]. Examining demographic, social, and environmental factors associated with *T. gondii* exposure in sentinel animals enables the implementation of preventive measures to reduce pathogen exposure within neighborhoods [19].

There is limited research on the prevalence and risk factors of *Toxoplasma gondii* infection in animal communities within informal urban settlements, despite the fact that these environments may present high levels of exposure and risk. Further research is needed to assess *T. gondii* epidemiology in local animal populations, in order to enhance understanding and improve infection control strategies in the region [26]. In particular, little is known about the dynamics of *T. gondii* infection in chickens under natural conditions [27], and studies rarely integrate data across multiple animal species. This study presents a comprehensive analysis encompassing companion animals (cats and dogs), domestic species (chicken and horses), wildlife (opossums), and synanthropic species (rats). By investigating such a diverse range of hosts within the same urban context, our research seeks to generate novel insights into the transmission dynamics and environmental circulation of *T. gondii* in vulnerable neighborhoods. Specifically, this study aims to assess the seroprevalence and identify associated risk factors for *T. gondii* infection in domestic and synanthropic animals from informal urban settlements in Salvador (BA, Brazil). These findings not only advance our scientific understanding of infection patterns but also provide valuable evidence to guide the development of targeted prevention and control strategies in comparable urban settings.

## Materials and methods

### Ethics statement

This research was approved by the Ethics Committee on the Use of Animals (CEUA) of the School of Veterinary Medicine and Animal Science at the Federal University of Bahia (protocol 07/2021), as well as by the Biodiversity Authorization and Information System (SISBIO, protocol 77314–1). Was also approved by the Research Ethics Committee of the Institute of Collective Health/Federal University of Bahia (CEP/ISC/UFBA) and by the National Commission for Ethics in Research (CONEP) linked to the Brazilian Ministry of Health under protocol number CAAE n° 35405320.0.1001.5030, approval opinion number 4.510.173/2021.

#### Study area.

**Fig 1:**
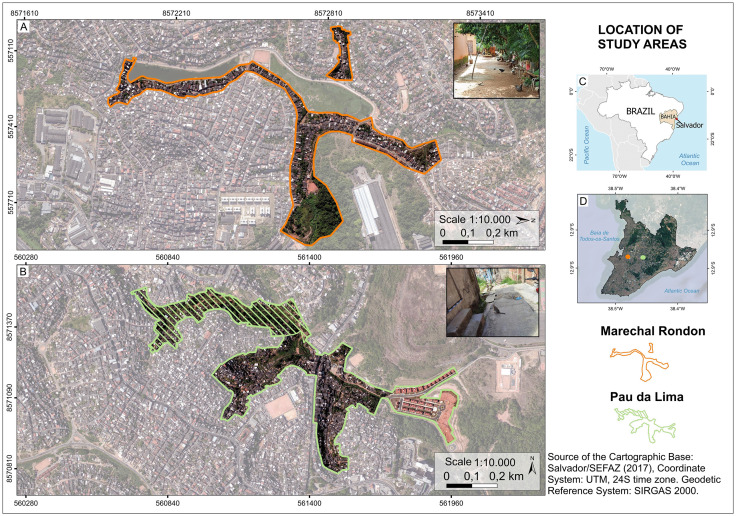
Study areas in the city of Salvador, Brazil. (A) The study area in the Marechal Rondon neighborhood is delineated by an orange line. (B) The study area in the Pau da Lima neighborhood is delineated by a green line. The area filled with diagonal lines represents the zone used for domestic animal sampling. (C) Map of Brazil showing the location of Salvador in the state of Bahia. (D) Map of Salvador (Bahia, Brazil) indicating the Marechal Rondon neighborhood with a green dot and Pau da Lima with an orange dot. Photos credit: L. Bazan. Source: Base image (Salvador/SEFAZ, 2017) [80]; Municipal and state limits (IBGE, 2017) [81].

A cross-sectional study was conducted within a Community-Based Participatory Research (CBPR) project to collect samples from animals between October 2021 and February 2023, parallel to a longitudinal epidemiological study in the same area [28]. This study was conducted in Salvador (Bahia, Brazil), located in the northeast of the country, with a population of 2,418,005 (IBGE, 2023). The eco-epidemiological research was conducted in two informal urban settlements in the Marechal Rondon and Pau da Lima neighborhoods. The total area of these neighborhoods is 0.65 and 1.15 km^2^, respectively. For the purpose of this research, the effective study area in each settlement was defined by sampling polygons measuring 0.16 km^2^ in Marechal Rondon and 0.46 km^2^ in Pau da Lima [29] (Fig 1). These neighborhoods were selected due to their shared characteristics typical of vulnerable urban areas: lack of urban planning, inadequate basic sanitation, and unfavorable socioeconomic conditions [30,31]. Although there are no previous studies on toxoplasmosis in animals or humans in these neighborhoods, several investigations have reported high prevalence rates of other neglected zoonotic diseases, such as leptospirosis [30,32] and hantavirus [33], highlighting the vulnerability of these areas.

#### Study design and sample collection.

The collection of biological samples and the recording of demographic and ecological information were conducted in two complementary phases throughout the sampling period. The first phase involved the collection of domestic animals (dogs, cats, chickens, and horses), while the second phase comprised the trapping of wild opossums (*Didelphis aurita*) and synanthropic rodents (*Rattus norvegicus*).

*Domestic animals sample collection:* All households within the polygons were identified with a unique code. Subsequently, all homes in the study area were visited, inviting domestic animal owners to participate in the research project. Blood samples from domestic animals were collected using a census approach, based on household records provided by residents who reported owning pets or other domestic animals in their homes and who signed an informed consent form authorizing the collection of biological samples from their animals. The animals were physically restrained to obtain general clinical data and to collect peripheral blood through cephalic or jugular venipuncture, ensuring that the collected volume did not exceed 1% of the animal’s body weight or a maximum of 5 mL of blood. Chemical restraint was selectively used for reactive cats [34]. Following the collection of blood samples, a semi-structured questionnaire was applied to the owner to assess epidemiological variables related to the clinical history, demographic information about the animal, and socio-environmental conditions of the home.

*Wild and synanthropic sample collection:* To ensure an adequate representation of the neighborhood, 114 and 107 evenly spaced random points were selected in Marechal Rondon and Pau da Lima, respectively, for sampling wild and synanthropic animals, with a minimum distance of 20 meters between capture points (Fig A in S1 Text). Two Tomahawk live traps baited with sausage and pineapple were deployed at each point for four nights, resulting in 910 attempted trap-nights in Marechal Rondon and 856 in Pau da Lima. This capture method was based on previous studies conducted in the same areas, which provided the basis for defining the sampling effort required [35,36]. The traps were checked every morning, and their baits replaced when necessary. The traps were situated in peri-domestic settings, such as backyards, following the acquisition of permission from the residents, and in public areas. For each sampling point, a questionnaire was administered by field personnel to assess the environmental characteristics of the peri-domestic area. Following their capture, the rats and opossums were transported to a field laboratory, where they were taxonomically identified, their body measurements were taken, and blood samples were collected using sterile vacuum blood collection tubes. For opossums, a maximum blood volume equivalent to 1% of the animal’s body weight was collected, whereas for rats, approximately 3–5 mL of blood was obtained, as these animals were euthanized for necropsy as part of other ongoing investigations. General clinical notes and overall physical condition were recorded. The captured opossums were anesthetized with ketamine (15mg/kg) and midazolam (0.25mg/kg), administered intramuscularly [37] prior to biological sample collection. The rats received ketamine (25 mg/kg) with xylazine (3 mg/kg) via intramuscular injection, and after blood collection by cardiac puncture, thiopental (100 mg/kg) was administered intraperitoneally for euthanasia. These sedation and euthanasia procedures for opossums and rats were conducted in accordance with the guidelines established by the CONCEA Normative Resolution N° 37 [38].

#### Serological testing.

Serum extraction was performed by centrifugation at 1811 x *g* for 15 minutes. Serum samples were aliquoted in 1.5 mL microtubes, identified, and stored at -20 °C until the serological test was performed. The anti-*T.gondii* IgG antibodies were detected using an indirect immunofluorescence antibody test (IFAT) with in-house RH strain tachyzoites obtained through cell culture, following the protocol described by Camargo (1964) with minor modifications [39]. No molecular or isolation confirmation was performed, as only blood samples were available for analysis; However, IFAT has shown to be a well-established and validated method for detecting *T. gondii* exposure in animals [40]. In dogs, the test presents a specificity of 95% and a sensitivity of 70% [41], while in cats, it reaches a specificity of 100% and a sensitivity of 98% [42]. Sera was diluted in phosphate-buffered saline (PBS). The cut-off point was 1:50, to minimize false-positive reactions that may occur at lower dilutions, based on previous studies that used similar criteria for animals [21,43–45]. Positive and negative controls of each species were used for each reaction. For the detection of IgG anti-*T. gondii* antibodies, were used commercial anti-Cat IgG and anti-Rat IgG produced in goats (Sigma-Aldrich) anti-Dog IgG, anti-Horse IgG, and anti-Chicken IgY produced in rabbit (Sigma-Aldrich) labeled with fluorescein isothiocyanate. The Zoonoses and Vector-borne Diseases Laboratory (DVZ, COVISA) produced the anti-opossum IgG in goats. The conjugates were diluted at 1:400 for dogs and chickens, 1:300 for cats, 1:100 for horses, and 1:50 for rats and opossums. These dilutions were determined based on preliminary tests using positive control samples.

#### Statistical analysis.

The seroprevalence was estimated as the percentage of individuals with a positive serological status on the IFAT, with a 95% confidence interval (CI). Prevalence differences among species were evaluated using the chi-square test. To investigate the best model to explain the serological status of *T. gondii*, generalized linear mixed models (GLMM) were applied with lme4 package [46]. The response variable, *T. gondii* seropositivity (positive/negative), was modeled assuming a binomial distribution with a logit link function. Prior to fitting the GLMMs, univariable generalized linear models (GLMs) were performed to explore the relationship between each demographic and environmental variable and the response variable. Those variables with a p-value < 0.2 were selected for inclusion in the full model. Household was included as a random variable, since animals sampled within the same household were exposed to the same owner-related and environmental conditions.

Model selection was based on Akaike Information Criterion (AIC) values using the “MuMIn” package [47]. The plausible models considered were those whose AIC difference was less than two compared to the best model. Among these, the final model was the most parsimonious, that is the one with the fewest explanatory variables. To analyze the collinearity between the variables, variance inflation factors were calculated, considering only those with a factor less than four. Model residuals were evaluated using the “DHARMa” package [48], which simulates standardized residuals for generalized linear (mixed) models. Residual diagnostics included tests for uniformity, dispersion, and outliers, as well as graphical inspection of simulated residual plots. All statistical analyses were performed using R Studio software (RStudio Team, 2020). Spatial distribution of seropositive and seronegative animals was visually described by the construction of maps using the geographic coordinates of the collection points in the QGIS version 3.26.0 program (QGIS Development Team, 2009).

In this study, we included a range of explanatory variables to evaluate factors associated with *T. gondii* seroprevalence in domestic and synanthropic animals within urban communities. For domestic animals, variables related to individual characteristics and environmental conditions were assessed. These included neighborhood, sex, sterilization status, age, type of diet, shelter type, management practices, vaccination, deworming status, household crowding (residents per room), garbage disposal practices, garbage deposit, access to paved areas, wall material, ground slope, activity of the Zoonosis Control Center (CCZ), backyard paving, presence of the peri-domestic regions, and frequency of garbage collection. For dogs, we excluded collinear variables, resulting in a complete model that retained neighborhood, sex, sterilization status, age, type of diet, shelter type, management practices, vaccination, deworming, residents per room, garbage deposit, paved access, ground slope, CCZ activity, backyard paving, peri-domestic areas, and garbage collection. For cats, the final model also excluded collinear variables, leaving neighborhood, age, type of diet, shelter type, management practices, vaccination, residents per room, garbage disposal practices, garbage deposit, wall material, ground slope, backyard paving, and garbage collection. For synanthropic animals, we included neighborhood, sex, age, body condition, and vegetation coverage.

## Results

**Fig 2:**
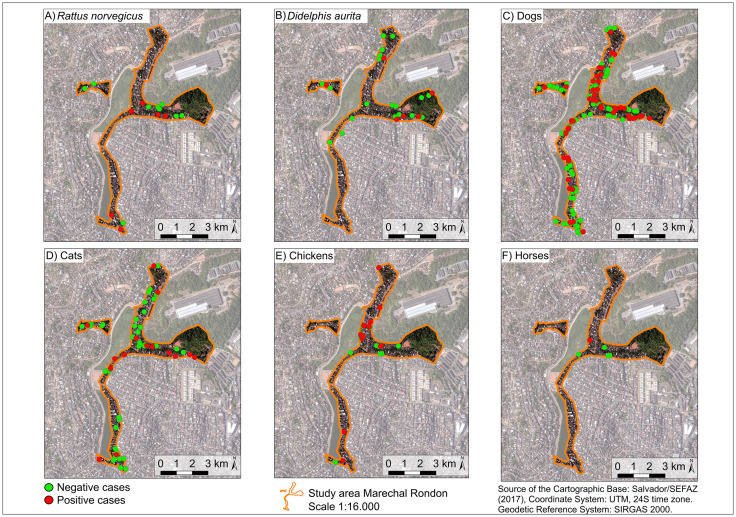
Spatial distribution of sampled individuals by species and frequency of *T. gondii*-seropositive animals in Marechal Rondon. Green points represent seronegative individuals and red points represent seropositive individuals. Source: Base image (Salvador/SEFAZ, 2017) [80].

**Fig 3:**
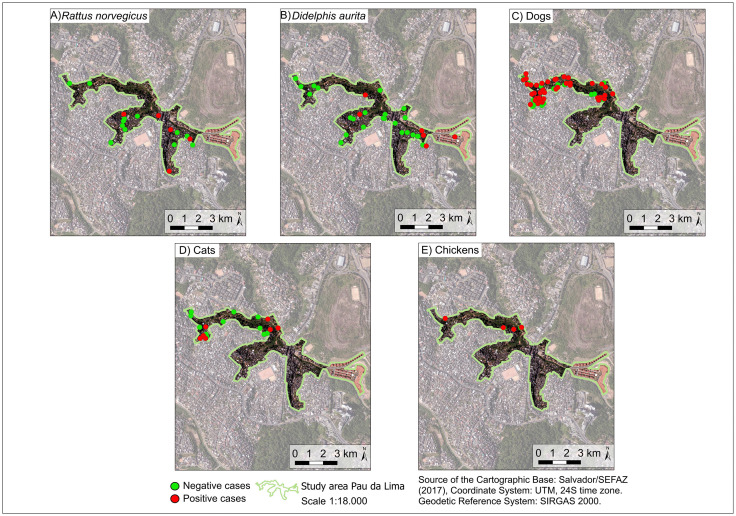
Spatial distribution of sampled individuals by species and frequency of *T. gondii*-seropositive animals in Pau da Lima. Green points represent seronegative individuals and red points represent seropositive individuals. Source: Base image (Salvador/SEFAZ, 2017) [80].

We collected blood samples from 288 dogs (*Canis lupus familiaris*), 112 cats (*Felis silvestris catus*), 27 chickens *(Gallus gallus domesticus*), six horses (*Equus ferus caballus*), 54 brown rats (*Rattus norvegicus*), and 75 big-eared opossums (*Didelphis aurita*). Of the total number of animals, 107/288 dogs (37.2%; 95% CI: 31.8 – 42.9), 25/112 cats (22.3%; 95% CI: 15.6 – 30.9), 18/27 chickens (66.7%; 95% CI: 47.8 – 81.4), 1/6 horses (16.7%; 95% CI: 0.42 – 64.1), 13/54 brown rats (24.1%; 95% CI: 14.6 – 37) and 15/75 big-eared opossums (20%; 95% CI: 12.5 – 30.4) were found to be seropositive for *T. gondii* (Figs 2, 3, Table 1, Tables A and B and C in S1 Text). A significant association was found between species and *T. gondii* seroprevalence status (Chi-square = 30.42, df = 5, *p* < 0.001), indicating that prevalence varied significantly among species.

**Table 1:** Frequency and seroprevalence of *Toxoplasma gondii* antibodies in animals from the Marechal Rondon and Pau da Lima neighborhoods.

Specie and neighborhood	Total	Positives	Prevalence (%)	95% CI
Dogs				
Marechal Rondon	173	53	30.6	23.9 – 38.1
Pau da Lima	115	54	47.0	37.6 – 56.5
Cats				
Marechal Rondon	86	18	20.9	12.9 – 31.0
Pau da Lima	26	7	26.9	11.6 – 47.8
Chickens				
Marechal Rondon	21	14	66.7	43.0 – 85.4
Pau da Lima	6	4	66.7	22.3 – 95.7
Horses				
Marechal Rondon	6	1	16.7	0.42 – 64.1
Pau da Lima	0	0	–	–
Brown rats				
Marechal Rondon	25	7	28.0	12.1 – 49.4
Pau da Lima	29	6	20.7	8.0 – 39.7
Big-eared opossums				
Marechal Rondon	36	8	22.2	10.1 – 39.1
Pau da Lima	39	7	17.9	7.5 – 33.5

In the final multiple regression model, the type of diet and neighborhood were associated with *T. gondii* seropositivity in dogs. In contrast, only the type of diet was associated with *T. gondii* seropositivity in cats. Dogs fed homemade food were almost six times as likely to be exposed to *T. gondii* (OR: 5.60; 95% CI 2.72 - 11.95) compared to dogs fed commercial food. Dogs fed a mixed diet were also found to be almost three times as likely to be exposed to *T. gondii* (OR: 2.82; 95% CI 1.50 - 5.52) compared to dogs fed commercial food. Dogs in Pau da Lima exhibited a twofold increased likelihood of exposure to *T. gondii* (OR: 1.95, 95% CI 1.17 - 3.26) compared to dogs in Marechal Rondon. In cats, consumption of homemade foods increased the probability of infection by *T. gondii* by fivefold (OR: 5; 95% CI 0.84 – 30.03), while consumption of mixed foods increased the likelihood of infection by *T. gondii* by twice (OR: 2.08; 95% CI 0.78 - 5.48) (Table 2). Residual diagnostics of both final models indicated no evidence of model misspecification. The simulated residuals were uniformly distributed, with no signs of overdispersion or outliers, confirming an adequate model fit.

**Table 2:** Multivariate analysis of the associated factors of *Toxoplasma gondii* seroprevalence in dogs and cats from Marechal Rondon and Pau da Lima, Salvador, BA, Brazil. OR: odds ratios; CI: 95% confidence interval; * Categories with a significant effect (p-value < 0.05).

Specie	Variables	OR	CI (95%)	*p*-value
Dogs	Type of diet*			
	Commercial (Ref.)	—	—	—
	Mixed	2.8	1.50 - 5.5	<0.01
	Homemade	5.6	2.72 - 11.9	<0.01
	Neighborhood*			
	Marechal Rondon (Ref.)	—	—	—
	Pau da Lima	1.9	1.17 - 3.3	0.01
Cats	Type of diet			
	Commercial (Ref.)	—	—	—
	Mixed	2.1	0.8 - 5.5	0.1
	Homemade	5	0.8 - 30.0	0.1

## Discussion

Our study allowed us to identify relevant variables, such as type of food and neighborhood, associated with *Toxoplasma gondii* infection in animals from socioeconomically vulnerable communities. The type of diet was identified as a common factor associated with *T. gondii* exposure in both dogs and cats. Our findings show that pets fed homemade diet had significantly higher odds of exposure to *T. gondii* than those fed commercial pet food. Consequently, commercial pet food diet acts as a protective factor against infection [49,50]. This observation aligns with the findings in São Paulo state that homemade feeding increases the odds of *T. gondii* exposure in dogs [51,52] and cats [53].

Because ingestion of food contaminated with oocysts is the main route of *T. gondii* infection in animals and humans [6], it is essential to consider the quality of homemade food provided to pets within the socioenvironmental context evaluated. Homemade diets may serve as potential sources of oocysts and contribute to the persistence of the parasite in urban environments [51,54]. In contrast, pets fed exclusively on commercial food are generally receive better nourished and health management, which reduces their 0susceptibility to infections [55,56]. Moreover, owners who feed their animals only commercial food often adopt additional appropriate management practices, such as restricting free roaming and maintaining regular vaccination and deworming schedules [57]. Together, hese factors further decrease the likelihood of exposure to *T. gondii* in the environment.

Furthermore, dogs from Pau da Lima were twice as likely to be exposed to *T. gondii* than those from Marechal Rondon. The higher seroprevalence observed in Pau da Lima may be associated with socio-environmental factors that increase dogs’ exposure to the pathogen. Variations could also influence differences between neighborhoods in the knowledge, attitudes, and practices of pet guardians [58], which warrants further investigation in future research. The seroprevalence of *T. gondii* in dogs in both neighborhoods was lower than the global seroprevalence of Brazil mentioned in Dubey et al. (2020), which is 70% [18]. This discrepancy is likely attributable to methodological differences as many studies cited used a lower cut-off point than the one employed in our research, potentially leading to inflated seroprevalence rates. In Marechal Rondon the seroprevalence was consistent with the 34% reported in dogs in Rio de Janeiro that presented for veterinary care, encompassing general check-ups and toxoplasmosis diagnosis [59] and comparable to the 30.7% prevalence seen in dogs from Curitiba that frequented densely occupied public spaces such as bus stations and parks [60].

In Pau da Lima the seroprevalence was similar to the 48% reported in domiciled dogs on Fernando de Noronha Island [61] and dogs from urban informal settlements in Jataizinho, Paraná [62]. By contrast, it was lower than the 70.5% reported in partly-domiciled dogs from Londrina, Paraná [63], where a lower cut-off point (1:16) was employed, likely contributing to the higher number of positives, and higher than the 9.5% reported in domiciled dogs from Garanhuns, Pernambuco [64].

The seroprevalence of *T. gondii* in cats in our study was comparable to the 21% observed in domiciled cats from Belém, Pará [65] and the 25% reported in cats from rural villages in the semi-arid region of Northeastern Brazil [66]. By contrast, it was lower than the global seroprevalence of 35% [67] and the 50% reported in pet cats of pregnant women attending healthcare services in Ilhéus, Bahia [68].

In chickens, the seroprevalence observer in our study exceeded the global prevalence of 30% [69] and was consistent with the 71% reported in free-range chickens from Minas Gerais [70]. Backyard chickens are considered a potential source for spreading this pathogen, as they are often raised for egg and meat consumption. Chickens play a significant role in the epidemiology of *T. gondii*, due to their clinical resistance to the parasite and the fact that cats fed naturally infected chicken tissues to shed millions of oocysts [71]. Poultry is also considered ideal sentinel species for monitoring environmental contamination with *T. gondii*, as their ground-feeding behavior exposes them directly to oocysts [21,27]. In urban informal settlements, chickens are frequently slaughtered at home or in unsupervised facilities, with viscera often left for scavengers or improperly discarded. *T. gondii* infection may occur if hygiene measures, after handling or cooking poultry, are not strictly followed. However, comprehensive risk assessment studies assessing the role of chickens in transmission dynamics within these settings remain limited.

The global seroprevalence of *T. gondii* antibodies in brown rats is 6%, while in South America is 18% [22], both lower than the 24% observed in this study. Due to their feeding behavior that predominantly facilitates oral transmission of sporulated oocysts, synanthropic rodents serve as indicators of environmental contamination. Consequently, the finding of *T. gondii* in rat populations might reflect the dissemination of the parasitic environmental phase within a specific area [23,72]. Rats are also recognized as reservoirs and important sources of infection for cats [73].

The seroprevalence of *T. gondii* in big-eared opossums observed here was comparable to the 22.7% reported in *Didelphis aurita* and *Didelphis albiventris* from urban areas in São Paulo state [74], and higher than the 5.5% reported in *Didelphis albiventris* also in São Paulo state [75]. Big-eared opossums can become infected by preying on infected rats [76,77], while simultaneously contributing to rat population control in urban neighborhoods. However, hunting opossums in peridomestic environments for human consumption or as food for domestic animals remains a significant factor that may substantially increase exposure to zoonotic pathogens, including *T. gondii* [78,79].

Due to the cross-sectional design and the use of serological techniques to detect antibodies, our study could not determine the timing of exposure to *T. gondii*. Additionally, the sample size of some of the analyzed species was too low, which may limit the accuracy for comparing the seroprevalence for all species and reduce the explanatory power of statistical models. Such variation in sampling could influence the generalizability of findings and the robustness of statistical associations drawn from the data. To overcome these limitations, we suggest that future studies incorporate environmental investigations assessing oocyst contamination in soil and water, as this would provide valuable insights into the parasite’s infection routes. At the same time, it would be important to continue studying *T. gondii* in other informal urban settlements of Salvador, allowing comparisons across different urban neighborhoods and contributing to a more comprehensive understanding of the parasite’s eco-epidemiology.

In the post–COVID-19 context, studying zoonotic diseases in historically neglected areas has become an urgent priority, particularly in the context of significant social and health inequalities in Brazil and around the world. Therefore, this study provides insights into the eco-epidemiology of *T. gondii* in urban animals, which could serve as sentinels for environmental contamination in vulnerable neighborhoods. The seroprevalence of *T. gondii* antibodies indicates exposure to the parasite and highlights a potential risk of infection within these neighborhoods. The finding that diet was identified as the main factor associated with *T. gondii* exposure in both dogs and cats reinforces the importance of promoting educational initiatives and campaigns in these neighborhoods to inform residents about the risks of infection for both animals and humans. Moreover, by linking parasite circulation with social vulnerability, poor sanitation, and local practices such as backyard poultry slaughter and opossum hunting, the study underscores the eco-social complexity of toxoplasmosis infection. This study holds significant relevance for public and animal health within a One Health perspective. By investigating *T. gondii* exposure across multiple domestic, synanthropic, and wild species in informal urban settlements, it highlights potential reservoirs and transmission pathways that may pose risks to humans, particularly in socially vulnerable communities with limited sanitation. These findings provide critical insights to guide prevention strategies, improve infection control, and support policies aimed at reducing zoonotic risks through integrated health and environmental interventions.

## Supporting information

S1 Text **Fig A.** Capture points (white dots) for wild animals in (A) Marechal Rondon and (B) Pau da Lima. Source: Base image (Salvador/SEFAZ, 2017) [80]. **Table A.** Univariate analysis of *T. gondii* in *Rattus norvegicus* and *Didelphis aurita*. * p-value < 0.05. **Table B.** Univariate analysis of *T. gondii* in dogs and cats. * p-value < 0.05. **Table C.** Univariate analysis of *T. gondii* in chickens and frequency of horses. * p-value < 0.05. **Table D.** Frequency of titers of *T. gondii* serology.
(DOCX)
