# Supplementary material for: Seroprevalence and risk factors for Toxoplasma gondii infection in wild, domestic and companion animals in urban informal settlements from Salvador, Brazil
Source: PLoS Negl Trop Dis. 2025 Dec 16;19(12):e0013303. doi: 10.1371/journal.pntd.0013303 (PMC12725734; doi:10.1371/journal.pntd.0013303)
Supplement: S1 Text — Fig A. Capture points (white dots) for wild animals in (A) Marechal Rondon and (B) Pau da Lima. Source: Base image (Salvador/SEFAZ, 2017) [80]. Table A. Univariate analysis of T. gondii in Rattus norvegicus and Didelphis aurita. * p-value < 0.05. Table B. Univariate analysis of T. gondii in dogs and cats. * p-value < 0.05. Table C. Univariate analysis of T. gondii in chickens and frequency of horses. * p-value < 0.05. Table D. Frequency of titers of T. gondii serology. (DOCX) [file pntd.0013303.s001.docx]

# Suplementary Information

**Seroprevalence and risk factors for *Toxoplasma gondii* infection in wild, domestic and companion animals in urban informal settlements from Salvador, Brazil**

Leonela Bazan^1^, Hernán Darío Argibay^2^, Waléria Borges-Silva^3^, Luís Fernando Pita Gondim^3^, Thaís Auxiliadora dos Santos Mattos^4^, Juliete Oliveira Santana^4^, Eduardo Mendes da Silva^1^, Michael Begon^6^, Hussein Khalil^5¶^, Federico Costa^1,2,7¶*^, Ianei de Oliveira Carneiro^3¶^

**Contents**

Fig A: Capture points (white dots) for wild animals in (A) Marechal Rondon and (B) Pau da Lima.


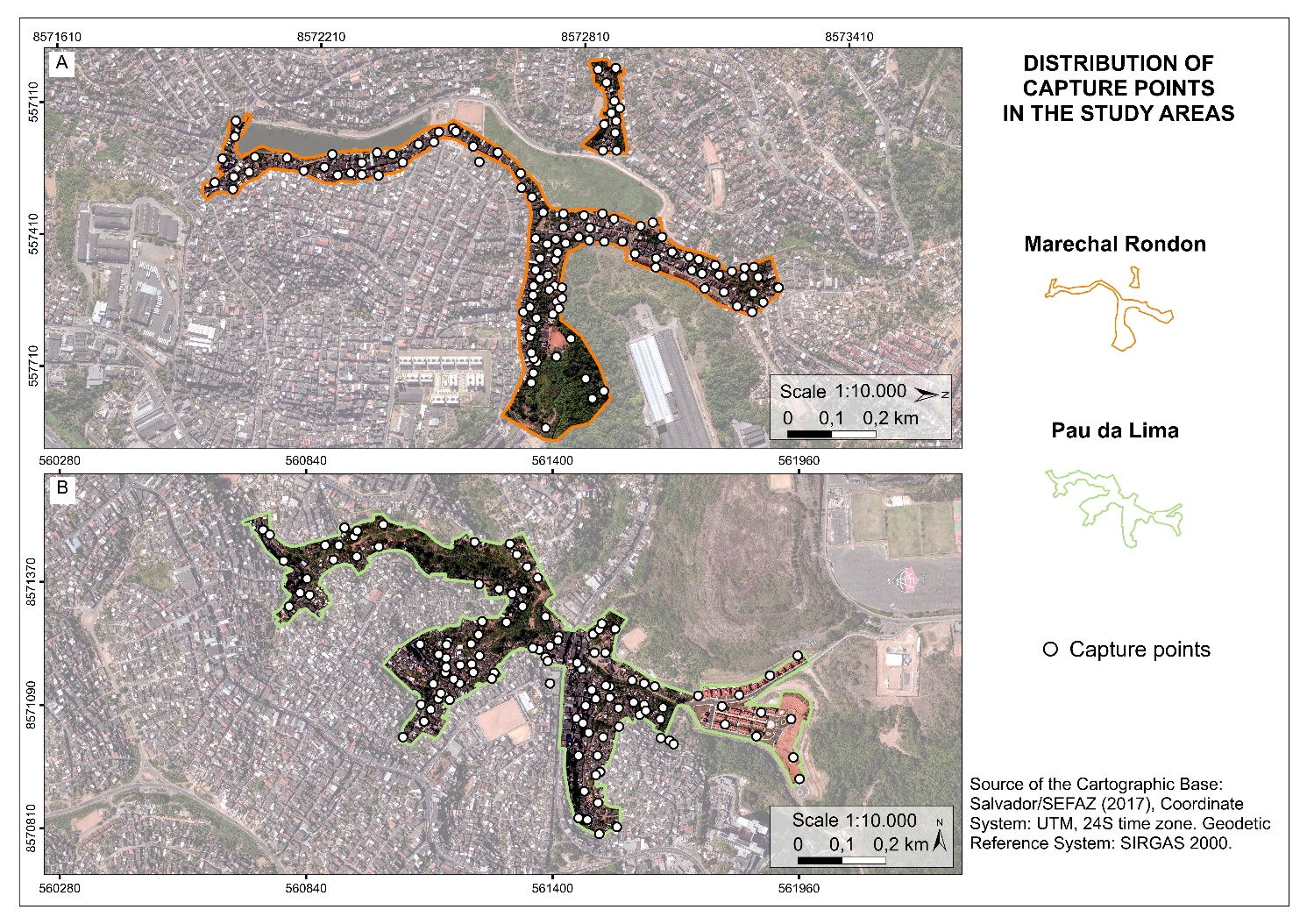


Table A: Univariate analysis of *T. gondii* in *Rattus norvegicus* and *Didelphis aurita.* * p-value < 0.05.

| **Variables and Categories** | ***Rattus norvegicus*** | | | | ***Didelphis aurita*** | | | |
| --- | --- | --- | --- | --- | --- | --- | --- | --- |
|  | **Total** | **Positives (%)** | **OR (95% CI)** | **p-value** | **Total** | **Positives (%)** | **OR (95% CI)** | **p-value** |
| Neighborhood |  |  |  |  |  |  |  |  |
| Marechal Rondon | 25 | 7 (28.0) |  |  | 32 | 7 (21.9) |  |  |
| Pau da Lima | 29 | 6 (20.7) | 0.67 (0.19 - 2.36) | 0.53 | 39 | 7 (17.9) | 0.78 (0.24 - 2.56) | 0.68 |
| Sex |  |  |  |  |  |  |  |  |
| Male | 23 | 5 (21.7) |  |  | 27 | 4 (14.8) |  |  |
| Female | 24 | 6 (25.0) | 1.20 (0.31 - 4.85) | 0.79 | 32 | 9 (28.1) | 2.25 (0.63 - 9.27) | 0.23 |
| NA | 7 | 2 (28.6) |  |  | 12 | 1 (8.3) |  |  |
| Age |  |  |  |  |  |  |  |  |
| Cub | - | - |  |  | 6 | 1 (16.7) |  |  |
| Juvenile | 14 | 1 (7.1) |  |  | 22 | 1 (4.5) | 0.24 (0.01 - 6.71) | 0.34 |
| Adult | 34 | 10 (29.4) | 5.42 (0.89 - 105) | 0.13 | 30 | 9 (30.0) | 2.14 (0.29 - 44.2) | 0.51 |
| NA | 6 | 2 (33.3) |  |  | 13 | 3 (23.1) |  |  |
| Body condition |  |  |  |  |  |  |  |  |
| Emaciated | - | - |  |  | 1 | 0 (0.0) |  |  |
| Thin | 2 | 1 (50.0) |  |  | 17 | 3 (17.6) |  |  |
| Normal | 38 | 9 (23.7) | 0.31 (0.01 - 8.38) | 0.42 | 37 | 8 (21.6) |  |  |
| Obese | 3 | 1 (33.3) | 0.50 (0.01 - 23.6) | 0.71 | - | - |  |  |
| NA | 11 | 2 (18.2) |  |  | 16 | 3 (18.8) |  |  |
| Urban coverage |  |  | 1.03 (1.0 - 1.07) | 0.10 |  |  | 0.99 (0.97 - 1.02) | 0.66 |
| Exposed soil |  |  | 1.11 (0.93 - 1.34) | 0.22 |  |  | 1.02 (0.83 - 1.21) | 0.82 |
| Vegetation |  |  | 0.96 (0.92 - 1.00) | 0.047* |  |  | 1.01 (0.98 - 1.05) | 0.41 |

Table B: Univariate analysis of *T. gondii* in dogs and cats. * p-value < 0.05.

|  | **Dogs** | | | | **Cats** | | | |
| --- | --- | --- | --- | --- | --- | --- | --- | --- |
| **Variables and categories** | **Total** | **Positives (%)** | **OR (95% CI)** | ***p*-value** | **Total** | **Positives (%)** | **OR (95% CI)** | ***p*-value** |
| Demographic variables | | | | | | | | |
| Sex |  |  |  |  |  |  |  |  |
| Male | 150 | 59 (39.33) |  |  | 71 | 14 (19.72) |  |  |
| Female | 138 | 48 (34.78) | 0.82 (0.51 - 1.33) | 0.42 | 41 | 11 (26.83) | 1.49 (0.60 - 3.69) | 0.39 |
| Age |  |  |  |  |  |  |  |  |
| Senile | 31 | 17 (54.84) |  |  | 4 | 1 (25) |  |  |
| Cub | 15 | 3 (20) | 0.38 (0.08 - 1.24) | 0.14 | 10 | 2 (20) | 0.97 (0.14 - 4.37) | 0.97 |
| Juvenile | 63 | 15 (23.81) | 0.47 (0.24 - 0.89) | 0.024* | 24 | 7 (29.17) | 1.59 (0.54 - 4.46) | 0.38 |
| Adult | 178 | 71 (39.89) | 1.83 (0.85 - 4.00) | 0.12 | 73 | 15 (20.55) | 1.29 (0.06 - 10.9) | 0.83 |
| NA | 1 | 1 (100) |  |  | 1 | 0 |  |  |
| Type of diet |  |  |  |  |  |  |  |  |
| Commercial food | 87 | 16 (18.39) |  |  | 72 | 12 (16.67) |  |  |
| Homemade food | 66 | 37 (56.06) | 5.66 (2.78 - 12.0) | <0.001* | 6 | 3 (50) | 5.00 (0.84 - 30.0) | 0.066 |
| Mixed | 135 | 54 (40) | 2.96 (1.58 - 5.76) | <0.001* | 34 | 10 (29.41) | 2.08 (0.78 - 5.48) | 0.14 |
| Shelter |  |  |  |  |  |  |  |  |
| Indoors | 108 | 37 (34.26) |  |  | 63 | 12 (19.05) |  |  |
| Peridomestic | 168 | 62 (36.9) | 1.12 (0.68 - 1.87) | 0.65 | 42 | 12 (28.57) | 1.70 (0.67 - 4.30) | 0.26 |
| Extradomiciliary | 10 | 6 (60) | 2.88 (0.77 - 11.9) | 0.12 | 7 | 1 (14.29) | 0.71 (0.04 - 4.71) | 0.76 |
| NA | 2 | 2 (100) |  |  | - | - |  |  |
| Handling |  |  |  |  |  |  |  |  |
| Domiciled | 167 | 53 (31.74) |  |  | 35 | 8 (22.86) |  |  |
| Semi-domiciled | 118 | 52 (44.07) | 1.69 (1.04 - 2.77) | 0.034* | 76 | 16 (21.05) | 0.90 (0.35 - 2.45) | 0.83 |
| NA | 3 | 2 (66.67) |  |  | 1 | 1 (100) |  |  |
| Sterilized |  |  |  |  |  |  |  |  |
| Yes | 24 | 9 (37.5) |  |  | 37 | 6 (16.22) |  |  |
| No | 255 | 91 (35.69) | 0.92 (0.40 - 2.28) | 0.86 | 73 | 17 (23.29) | 1.57 (0.58 - 4.72) | 0.39 |
| NA | 9 | 7 (77.78) |  |  | 2 | 2 (100) |  |  |
| Vaccination |  |  |  |  |  |  |  |  |
| Yes | 218 | 78 (35.78) |  |  | 71 | 14 (19.72) |  |  |
| No | 66 | 25 (37.88) | 1.09 (0.61 - 1.92) | 0.76 | 41 | 11 (26.83) | 1.49 (0.60 - 3.69) | 0.39 |
| NA | 4 | 4 (100) |  |  | - | - |  |  |
| Deworming |  |  |  |  |  |  |  |  |
| Yes | 190 | 55 (28.95) |  |  | 59 | 15 (25.42) |  |  |
| No | 94 | 48 (51.06) | 2.56 (1.54 - 4.29) | <0.001* | 53 | 10 (18.87) | 0.68 (0.27 - 1.67) | 0.41 |
| NA | 4 | 4 (100) |  |  | - | - |  |  |
| Environmental variables | | | | | | | | |
| Neighborhood |  |  |  |  |  |  |  |  |
| Marechal Rondon | 173 | 53 (30.64) |  |  | 86 | 18 (20.93) |  |  |
| Pau da Lima | 115 | 54 (46.96) | 2.00 (1.23 - 3.28) | 0.005* | 26 | 7 (26.92) | 1.39 (0.48 - 3.73) | 0.52 |
| Paved access |  |  |  |  |  |  |  |  |
| Yes | 208 | 72 (34.62) |  |  | 85 | 14 (16.47) |  |  |
| No | 65 | 28 (43.08) | 1.43 (0.81 - 2.52) | 0.22 | 23 | 8 (34.78) | 2.70 (0.94 - 7.56) | 0.059 |
| NA | 15 | 7 (46.67) |  |  | 4 | 3 (75) |  |  |
| Peridomestic área |  |  |  |  |  |  |  |  |
| No | 58 | 13 (22.41) |  |  | 29 | 7 (24.14) |  |  |
| Yes | 212 | 85 (40.09) | 2.32 (1.21 - 4.71) | 0.015* | 77 | 14 (18.18) | 0.70 (0.25 - 2.05) | 0.49 |
| NA | 18 | 9 (50) |  |  | 6 | 4 (66.67) |  |  |
| Backyard with paving |  |  |  |  |  |  |  |  |
| Yes | 102 | 39 (38.24) |  |  | 41 | 5 (12.2) |  |  |
| No - backyard with dirt/grass | 81 | 37 (45.68) | 1.36 (0.75 - 2.46) | 0.31 | 36 | 10 (27.78) | 2.77 (0.88 - 9.80) | 0.092 |
| No backyard | 86 | 22 (25.58) | 0.56 (0.29 - 1.03) | 0.066 | 29 | 6 (20.69) | 1.88 (0.51 - 7.21) | 0.34 |
| NA | 19 | 9 (47.37) |  |  | 6 | 4 (66.67) |  |  |
| Slope |  |  |  |  |  |  |  |  |
| No | 211 | 67 (31.75) |  |  | 90 | 19 (21.11) |  |  |
| Yes | 60 | 31 (51.67) | 2.30 (1.28 - 4.13) | 0.005* | 17 | 3 (17.65) | 0.80 (0.17 - 2.77) | 0.75 |
| NA | 17 | 9 (52.94) |  |  | 5 | 3 (60) |  |  |
| Wall material |  |  |  |  |  |  |  |  |
| Exposed brick | 26 | 11 (42.31) |  |  | 14 | 5 (35.71) |  |  |
| Concrete or covered brick | 243 | 86 (35.39) | 1.85 (0.07 - 47.1) | 0.67 | 94 | 17 (18.09) |  |  |
| Wood or other similar non-masonry material | 1 | 1 (100) | 1.58 (0.69 - 3.58) | 0.27 |  |  | 2.52 (0.70 - 8.29) | 0.14 |
| NA | 18 | 9 (50) |  |  | 4 | 3 (75) |  |  |
| CCZ activity |  |  |  |  |  |  |  |  |
| Months | 112 | 35 (31.25) |  |  | 49 | 10 (20.41) |  |  |
| More than a year | 83 | 34 (40.96) | 1.53 (0.84 - 2.77) | 0.16 | 28 | 4 (14.29) | 0.65 (0.16 - 2.19) | 0.50* |
| Never | 69 | 27 (39.13) | 1.41 (0.75 - 2.65) | 0.28 | 26 | 7 (26.92) | 1.44 (0.46 - 4.35) | 0.52 |
| NA | 24 | 11 (45.83) |  |  | 9 | 4 (44.44) |  |  |
| Deposit garbage |  |  |  |  |  |  |  |  |
| Empty lot | 18 | 4 (22.22) |  |  | 8 | 1 (12.5) |  |  |
| Right in front of the house | 53 | 18 (33.96) | 0.83 (0.43 - 1.56) | 0.58 | 19 | 7 (36.84) | 2.96 (0.95 - 8.92) | 0.054 |
| Hanging | 1 | 1 (100) | 0.46 (0.13 - 1.35) | 0.19 | - | - |  |  |
| Community bin | 194 | 74 (38.14) | 3.24 (0.31 - 70.5) | 0.34 | 79 | 13 (16.46) | 5.08 (0.19 - 134) | 0.26 |
| Others | 3 | 2 (66.67) |  |  | 2 | 1 (50) |  |  |
| NA | 19 | 9 (47.37) |  |  | 4 | 3 (75) |  |  |
| Garbage disposal |  |  |  |  |  |  |  |  |
| Plastic bags only | 251 | 90 (35.86) |  |  | 92 | 17 (18.48) |  |  |
| Only containers with lids | 6 | 2 (33.33) | 0.89 (0.12 - 4.68) | 0.90 | 4 | 1 (25) | 1.47 (0.07 - 12.3) | 0.74 |
| Both | 10 | 5 (50) | 1.79 (0.49 - 6.59) | 0.37 | 7 | 3 (42.86) | 3.31 (0.61 - 16.4) | 0.14 |
| Others | 3 | 1 (33.33) | 0.89 (0.04 - 9.46) | 0.93 | 5 | 1 (20) | 1.10 (0.05 - 8.06) | 0.93 |
| NA | 18 | 9 (50) |  |  | 4 | 3 (75) |  |  |
| Garbage collection |  |  |  |  |  |  |  |  |
| Daily | 209 | 73 (34.93) |  |  | 85 | 17 (20) |  |  |
| Once a week | 21 | 9 (42.86) | 1.40 (0.55 - 3.46) | 0.47 | 12 | 2 (16.67) | 0.80 (0.12 - 3.41) | 0.79 |
| Twice a week | 21 | 9 (42.86) | 1.40 (0.55 - 3.46) | 0.47 | 5 | 1 (20) | 1.00 (0.05 - 7.32) | >0.99 |
| Three times a week | 14 | 5 (35.71) | 1.04 (0.31 - 3.11) | 0.95 | 4 | 1 (25) | 1.33 (0.06 - 11.2) | 0.81 |
| NA | 23 | 11 (47.83) |  |  | 6 | 4 (66.67) |  |  |
| Residents per room |  |  | 1.13 (0.86 - 1.49) | 0.38 |  |  | 1.54 (0.95 - 2.48) | 0.075 |

Table C Univariate analysis of *T. gondii* in chickens and frequency of horses. * p-value < 0.05.

|  | **Chickens** | | **Horses** | |
| --- | --- | --- | --- | --- |
| **Variables and categories** | **Total** | **Positives (%)** | **Total** | **Positives (%)** |
| Demographic variables | | | | |
| Sex |  |  |  |  |
| Female | 22 | 16 (72.73) | 4 | 0 |
| Male | 5 | 2 (40) | 2 | 1 (50) |
| Age |  |  |  |  |
| Cub | - | - | 1 | 1 (100) |
| Juvenile | 4 | 4 (100) | 3 | 0 |
| Adult | 21 | 17 (80.95) | 2 | 0 |
| NA | 2 | 1 (50) | - |  |
| Type of diet |  |  |  |  |
| Commercial food | 3 | 1 (33.33) | - | - |
| Homemade food | 1 | 0 | - | - |
| Mixed | 23 | 17 (73.91) | 6 | 1 (16.67) |
| Shelter |  |  |  |  |
| Indoors | - | - | 4 | 0 |
| Peridomestic | 24 | 16 (66.67) | - | - |
| Extradomiciliary | 2 | 2 (100) | 2 | 1 (50) |
| NA | 1 | 0 | - | - |
| Handling |  |  |  |  |
| Domiciled | 8 | 5 (62.5) | 4 | 0 |
| Semi-domiciled | 12 | 7 (58.33) | 2 | 1 (50) |
| NA | 7 | 6 (85.71) |  |  |
| Sterilized |  |  |  |  |
| No | 23 | 14 (60.87) | 3 | 1 (33.33) |
| Yes | - | - | 2 | 0 |
| NA | 4 | 4 (100) | 1 | 0 |
| Vaccination |  |  |  |  |
| No | 26 | 18 (69.23) | 3 | 1 (33.33) |
| Yes | - | - | 3 | 0 |
| NA | 1 | 0 |  |  |
| Deworming |  |  |  |  |
| No | 2 | 0 | - | - |
| Yes | 2 | 2 (100) | 1 | 1 (100) |
| NA | - | - | 5 | 0 |
| Neighborhood |  |  |  |  |
| Marechal Rondon | 21 | 14 (66.67) | 6 | 1 (16.67) |
| Pau da Lima | 6 | 4 (66.67) | - | - |
| Paved access |  |  |  |  |
| No | 6 | 4 (66.67) | - |  |
| Yes | 17 | 11 (64.71) | 5 | 1 (20) |
| NA | 4 | 3 (75) | 1 | 0 |
| Peridomestic area |  |  |  |  |
| No | - | - | 1 | 0 |
| Yes | 23 | 15 (65.22) | 3 | 0 |
| NA | 4 | 3 (75) | 2 | 1 (50) |
| Backyard with paving |  |  |  |  |
| No - backyard with dirt/grass | 12 | 7 (58.33) | 1 | 0 |
| Yes | 7 | 6 (85.71) | 3 | 0 |
| No backyard | 4 | 2 (50) | - | - |
| NA | 4 | 3 (75) | 2 | 1 (50) |
| Slope |  |  |  |  |
| No | 17 | 11 (64.71) | 4 | 0 |
| Yes | 6 | 4 (66.67) | - | - |
| NA | 4 | 3 (75) | 2 | 1 (50) |
| Wall material |  |  |  |  |
| Wood or other similar non-masonry material | 4 | 2 (50) | - |  |
| Concrete or covered brick | 2 | 1 (50) | 4 | 0 |
| Exposed brick | 17 | 12 (70.59) |  |  |
| NA | 4 | 3 (75) | 2 | 1 (50) |
| CCZ activity |  |  |  |  |
| Months | 8 | 6 (75) |  |  |
| More than a year | 7 | 4 (57.14) | 3 | 0 |
| Never | 8 | 5 (62.5) | 1 | 0 |
| NA | 4 | 3 (75) | 2 | 1 (50) |
| Deposit garbage |  |  |  |  |
| Right in front of the house | 2 | 2 (100) | 2 | 0 |
| Community bin | 17 | 11 (64.71) | 2 | 0 |
| Empty lot | 2 | 1 (50) | - | - |
| Others | 2 | 1 (50) | - | - |
| NA | 4 | 3 (75) | 2 | 1 (50) |
| Garbage disposal |  |  |  |  |
| Only containers with lids |  |  |  |  |
| Plastic bags only | 16 | 10 (62.5) | 4 | 0 |
| Both | 5 | 4 (80) | - | - |
| Others | 2 | 1 (50) | - | - |
| NA | 4 | 3 (75) | 2 | 1 (50) |
| Garbage collection |  |  |  |  |
| Daily | 18 | 12 (66.67) | 4 | 0 |
| Once a week | 2 | 1 (50) | - | - |
| Twice a week | 2 | 1 (50) | - | - |
| Three times a week | 1 | 1 (100) | - | - |
| NA | 4 | 3 (75) | 2 | 1 (50) |
| Residents per room |  |  |  |  |

Table D: Frequency of titers of *T. gondii* serology

|  | 1:50 | | 1:100 | | 1:200 | | 1:400 | | 1:800 | | Total | |
| --- | --- | --- | --- | --- | --- | --- | --- | --- | --- | --- | --- | --- |
| **Species** | MR (%) | PDL (%) | MR (%) | PDL (%) | MR (%) | PDL (%) | MR (%) | PDL (%) | MR (%) | PDL (%) | **MR** | **PDL** |
| **Dogs** | 8 (15.1) | 6 (11.1) | 21 (39.6) | 13 (24.1) | 16 (30.2) | 11 (20.4) | 4 (7.5) | 12 (22.2) | 4 (7.5) | 12 (22.2) | **53** | **54** |
| **Cats** | - | - | 1 (5.6) | - | 2 (11.1) | - | - | 1 (12.5) | 15 (83.3) | 7 (87.5) | **18** | **8** |
| **Chickens** | 1 (7.1) | - | 1 (7.1) | - | 3 (21.4) | - | 5 (35.7) | - | 4 (28.6) | 4 (100) | **14** | **4** |
| **Horses** | - | - | - | - | 1 (100) | - | - | - | - | - | **1** | **-** |
| **Brown rats** | 1 (14.3) | 2 (33.3) | 1 (14.3) | 1 (16.7) | 2 (28.6) | 2 (33.3) | 1 (14.3) | - | 2 (28.6) | 1 (16.7) | **7** | **6** |
| **Big-eared opossums** | 1 | 1 (14.3) | 2 (28.6) | 2 (28.6) | 3 (42.9) | 1 (14.3) | - | 1 (14.3) | 1 (14.3) | 2 (28.6) | **7** | **7** |
| **TOTAL** | **11 (11)** | **9 (11.4)** | **26 (26)** | **16 (20.3)** | **27 (27)** | **14 (17.7)** | **10 (10)** | **14 (17.7)** | **26 (26.0)** | **26 (32.9)** | **100** | **79** |

(MR: Marechal Rondon; PDL: Pau da Lima
